# Opossum carboxylesterases: sequences, phylogeny and evidence for *CES *gene duplication events predating the marsupial-eutherian common ancestor

**DOI:** 10.1186/1471-2148-8-54

**Published:** 2008-02-20

**Authors:** Roger S Holmes, Jeannie Chan, Laura A Cox, William J Murphy, John L VandeBerg

**Affiliations:** 1 Department of Genetics, Southwest Foundation for Biomedical Research, San Antonio, TX, USA; 2 Southwest National Primate Research Center, Southwest Foundation for Biomedical Research, San Antonio, TX, USA; 3 School of Biomolecular and Physical Sciences, Griffith University, Nathan, Queensland, Australia; 4 Department of Veterinary Integrative Biosciences, College of Veterinary Medicine and Biomedical Sciences, Texas A&M University, College Station, TX, USA

## Abstract

**Background:**

Carboxylesterases (CES) perform diverse metabolic roles in mammalian organisms in the detoxification of a broad range of drugs and xenobiotics and may also serve in specific roles in lipid, cholesterol, pheromone and lung surfactant metabolism. Five CES families have been reported in mammals with human CES1 and CES2 the most extensively studied. Here we describe the genetics, expression and phylogeny of CES isozymes in the opossum and report on the sequences and locations of *CES1*, *CES2 *and *CES6 *'like' genes within two gene clusters on chromosome one. We also discuss the likely sequence of gene duplication events generating multiple *CES *genes during vertebrate evolution.

**Results:**

We report a cDNA sequence for an opossum *CES *and present evidence for *CES1 *and *CES2 *like genes expressed in opossum liver and intestine and for distinct gene locations of five opossum *CES *genes,*CES1*, *CES2.1*, *CES2.2*, *CES2.3 *and *CES6*, on chromosome 1. Phylogenetic and sequence alignment studies compared the predicted amino acid sequences for opossum CES with those for human, mouse, chicken, frog, salmon and *Drosophila *CES gene products. Phylogenetic analyses produced congruent phylogenetic trees depicting a rapid early diversification into at least five distinct CES gene family clusters: *CES2, CES1, CES7, CES3*, and *CES6*. Molecular divergence estimates based on a Bayesian relaxed clock approach revealed an origin for the five mammalian CES gene families between 328–378 MYA.

**Conclusion:**

The deduced amino acid sequence for an opossum cDNA was consistent with its identity as a mammalian *CES2 *gene product (designated *CES2.1*). Distinct gene locations for opossum *CES1 *(1: 446,222,550–446,274,850), three *CES2 *genes (1: 677,773,395–677,927,030) and a *CES6 *gene (1: 677,585,520–677,730,419) were observed on chromosome 1. Opossum *CES1 *and multiple *CES2 *genes were expressed in liver and intestine. Amino acid sequences for opossum *CES1 *and three *CES2 *gene products revealed conserved residues previously reported for human CES1 involved in catalysis, ligand binding, tertiary structure and organelle localization. Phylogenetic studies indicated the gene duplication events which generated ancestral mammalian *CES *genes predated the common ancestor for marsupial and eutherian mammals, and appear to coincide with the early diversification of tetrapods.

## Background

Carboxylesterases (CES; E.C.3.1.1.1) catalyse many hydrolytic and transesterification reactions and use a wide range of substrates, including xenobiotic carboxyl esters, thioesters and aromatic amides, anticancer drugs such as CPT-11 and capecitabine, narcotics such as heroine and cocaine, clinical drugs such as lovastatin and lidocaine, organophosphate and carbamate poisons such as sarin, tabun and soman and insecticides (eg. malathion) [1-3]. The enzyme also catalyses reactions in cholesterol and fatty acid metabolism, including fatty acyl CoA hydrolase [4], acyl CoA: cholesterol acyl transferase [5], cholesterol:ester hydrolase [6], acyl carnitine hydrolase [7], fatty acyl: ethyl ester synthase [8] and triacylglycerol hydrolase [9], and may serve specific roles in lung surfactant [10] and pheromone [11] metabolism. CES is predominantly localized in the endoplasmic reticulum (ER) and has an N-terminal hydrophobic signal peptide consistent with a trafficking role through the ER [12].

CES is widely distributed in biological organisms and has been extensively studied in mammals, particularly in humans, mouse and rat [7,13-15]. Six human CES genes have been reported on chromosome 16: *CES1*, encoding the major liver enzyme which is also found in lung epithelia, macrophages and other tissues [16,17]; *CES2*, encoding the major intestinal enzyme and also expressed in liver, kidney, heart and skeletal muscle [18,19]; *CES3*, expressed in liver, colon and small intestine [20,21]; and *CES4 *[22,23], *CES6 *[24] and *CES7 *[25,26], the products of which are less well characterized as proteins. The genes occur in 2 clusters approximately 11 million bps apart on chromosome 16: *CES4.CES1.CES7 *and *CES2.CES3.CES6 *[RS Holmes, J Glenn, JL VandeBerg & LA Cox: Baboon carboxylesterases 1 and 2: sequences, structures and phylogenetic relationships with human and other primate carboxylesterases, unpublished]. The close proximity and sequence similarity of these genes (47% identity for human *CES1 *and *CES2 *cDNAs) imply that they arose from an ancestral gene duplication event [27,28]. Tertiary and quaternary structures for several human CES1 complexes have been determined at high resolution (2.8Å) which are consistent with three functional domains: the catalytic domain containing the active site 'triad' and the carbohydrate binding site; the αβ domain supporting the majority of the hydrophobic internal structure and the subunit-subunit binding sites; and the regulatory domain which facilitates substrate binding, product release and the trimer-hexamer equilibrium [3,15,29,30].

Studies on the evolution of CES have demonstrated that genetic variability for this enzyme contributes significantly to selection, particularly for *Drosophila*, where multiple genes have been identified, including EST6, which plays a key role in reproduction, and JHE (juvenile hormone esterase), which serves an essential role in insect development [31,32]. In addition, evolutionary selection mechanisms for insecticide resistance have been observed in several arthropod species by the amplification of CES genes [33]. Previous studies on mammalian CES evolution have focused on the identification and classification of multiple CES genes, particularly for human, mouse, rat and other eutherian mammals [1,2,7,18,22,28]. Initial studies described two major mammalian CES gene families, CES1 and CES2, which apparently evolved from an ancestral CES gene [16,18,28], whereas more recent reports have proposed five CES gene families, based on phylogenetic analyses of 48 sequences of mammalian CES [7], which are consistent with the five families of human CES genes described earlier [16-26].

This paper extends current knowledge on CES evolution to a marsupial species and reports the cDNA and deduced amino acid sequence for an opossum CES (designated as CES2.1), RT-PCR expression and *in silico *studies providing evidence for *CES1 *and *CES2 *'like' genes on chromosome 1 of the opossum and the phylogenetic relationships of opossum CES2.1 and predicted opossum CES gene products with human CESs. The opossum is a marsupial which is used as an animal model in studying the genetics of heart disease and cancer [34,35], and given the roles of mammalian CES in cholesterol, fatty acid and xenobiotic metabolism, these studies may contribute significantly to the identification of candidate genes for these diseases. In addition, this report on opossum CES provides evidence for the appearance of five CES gene families prior to metatherian mammals during vertebrate evolution.

## Results and discussion

### Opossum *CES2 *Gene Products

The open reading frame of the full-length cDNA (GenBank: EU019537) comprised 1653 nucleotides; and the 5' UTR and 3' UTR comprised 28 nucleotides and 99 nucleotides, respectively. This was obtained by using primers based on a 421 bp opossum cDNA (GenBank: DR038241) showing sequence similarity to mammalian CES to amplify a full-length opossum *CES *cDNA from liver RNA. The best-scoring alignment in a BLAST analysis [36,37] for the opossum cDNA sequence was with a human CES2 sequence, and the opossum cDNA is referred to as CES2.1 (gene designation: *CES2.1*). Using the opossum *CES2 *cDNA as the query in a BLAT search [38,39], we found two additional CES gene regions and cDNAs which are referred to as CES2.2 (gene designation: *CES2.2*) and CES2.3 (gene designation: *CES2.3) *(Table 1). These latter genes were subjected to further analyses using RT-PCR and sequencing the product *CES *cDNAs.

**Table 1:** Carboxylesterase (CES) Genes and Enzymes Examined

Organism	CES Gene	GenBank	CES	UNIPROT	Amino	Chromosome: Location
		ID	Lineage	ID	Acids	derived from BLAT studies
Human	CES1	L07765	CES1	P23141	567	16: 54,394,266–54,424,494
	CES2	BX538086	CES2	O00248	559	16: 65,525,828–65,536,493
	CES3	AK025389	CES3	Q6UWW8	571	16: 65,558,879–65,566,552
	CES6	AY358504	CES6	Q6UX55	561	16: 65,580,134–65,601,160
	CES7	FLJ31547	CES7	Q96DN9	525	16: 54,437,629–54,466,783
Mouse	CES3	NM053200	CES1	Q8VCT4	565	8:95,690,157–95,721,618
	CES2	NM145603	CES2	Q91WG0	561	8:107,371,033–107,378,161
	CES31	BC061004	CES3	Q63880	568	8:107,572,572–107,582,000
	CES8	BC028374	CES6	N/A	563	8:107,655,852–107,673,417
	CES7	XM146444	CES7	Q6AW46	575	8: 96038096–96059607
Opossum	CES1	EU074630	CES1	N/A	559	1: 446,222,550–446,274,850
	CES2.1	EU019537	CES2	N/A	550	1: 677,900,820–677,927,030
	CES2.2	EU019538	CES2	N/A	562	1: 677,773,395–677,808,444
	CES2.3	EU019539	CES2	N/A	562	1: 677,826,454–677,852,862
	CES6	N/A	CES6	N/A	592	1: 677,585,520–677,730,419
Chicken	CES1	BX929672	CES1	N/A	442	11:11,992,177–12,025,392
Frog	CES	BC082503	CES2	N/A	557	scaffold_170:71,809–89,385
Salmon	CES	Z25867	CES	Q92149	540	N/A
Fly	EST6	J04167	CES	P08171	544	3L:12181935–12183681
	ESTP	M33780	CES	P18167	544	3L:12183838–12185528

### Opossum CES1 and CES2 Gene Expression Studies

**Figure 1:**
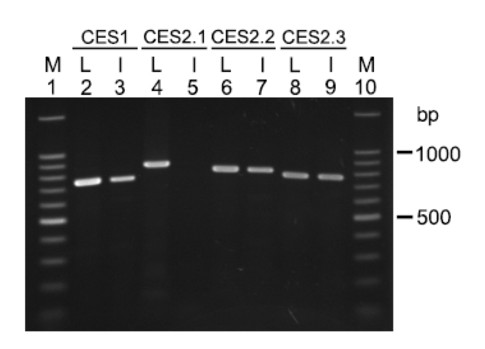
**Expression of *CES *genes in opossum.** Liver and intestinal cDNAs were reverse transcribed from DNase I-treated RNA, and they were used as templates in RT-PCR to analyze *CES *gene expression. Lanes 2 and 3 are RT-PCR products amplified from liver (L) and intestine (I) cDNAs for the *CES1 *gene; lanes 4 and 5, RT-PCR products from liver (L) and intestine (I) cDNAs for the *CES2.1 *gene; lanes 6 and 7, RT-PCR products from liver (L) and intestine (I) for cDNAs for the *CES2.2 *gene; and lanes 8 and 9, RT-PCR products from liver (L) and intestine (I) for cDNAs for the *CES2.3 *gene. M shows the DNA size ladder.

Expression of the predicted *CES *genes in the liver and small intestine was analyzed by RT-PCR (Figure 1). Controls were also conducted to ensure that DNase-treated liver and intestine RNAs could not serve as templates for RT- PCR (See Additional File 1).

An opossum *CES1 *gene was identified by *in silico *methods using the human CES1 amino acid sequence to interrogate the opossum genome [40] in a BLAT analysis [37-39]. Primers based on the predicted opossum *CES1 *cDNA sequence amplified a 750 bp PCR product which was similar to the predicted PCR product (741 bp), indicating that the *CES1 *gene is expressed in liver and intestine.

Unlike the *CES1 *gene, the *CES2.1 *gene was expressed in the liver, but not in the small intestine. The *CES2.2 *and *CES2.3 *genes were also identified by *in silico *methods. Primers for the *CES2.2 *cDNA amplified an 800 bp PCR product, which was similar to the predicted PCR product (803 bp), indicating that the *CES2.2 *gene is expressed in liver and intestine. However, primers for *CES2.3 *cDNA amplified a PCR product of about 770 bp, which is larger than the 667 bp PCR product based on the predicted cDNA sequence. The *CES2.3 *cDNA was sequenced (GenBank: EU019539) and this showed that 99 nucleotides were missing from the predicted BLAT derived sequence. The PCR product for the *CES2.3 *gene should have been 766 bp. These results indicate that the *CES2.3 *gene is expressed in both liver and small intestine and that the BLAT derived sequence contained a deletion as compared with the actual sequence for the *CES2.3 *RT-PCR product. We also sequenced the *CES2.2 *cDNA (Accession No. EU019538), and found that 33 nucleotides in the predicted sequence were absent in the *CES2.2 *cDNA sequence. These RT-PCR studies of opossum *CES2.2 *and *CES2.3 *genes show that the BLAT software [38,39] used to predict sequences for opossum CES gene products did not fully recognize all of the intron-exon junctions resulting in some deletions or insertions that are not found in the *in vivo *gene product. This emphasizes the importance of undertaking cDNA sequencing to obtain sequences for these and other cDNAs.

### Opossum CES2.1, CES2.2 and CES2.3 Deduced Amino Acid Sequences

**Figure 2:**
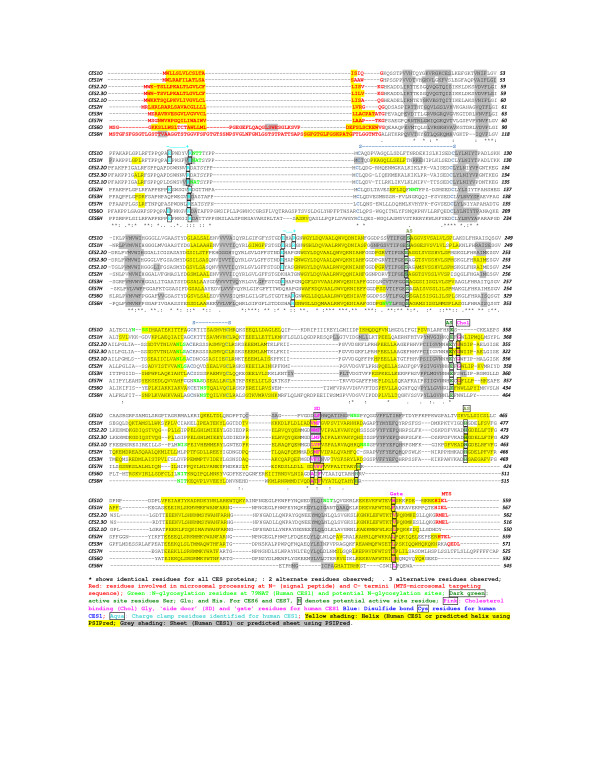
**Alignments of human CES1 (***CES1H***), CES2 ***(CES2H)***, CES3 ***(CES3H)***, CES6 ***(CES6H) ***and CES7 ***(CES7H) ***amino acid sequences with predicted opossum proteins: CES1 ***(CES1O)***; CES2.1 ***(CES2.1O)***, CES2.2 ***(CES2.2O)***, CES2.3 ***(CES2.3O) ***and CES6 ***(CES6O)***.**

The deduced amino acid sequence for opossum CES2.1 is shown in Figure 2 with the previously reported sequence for human CES2 [18,19], together with the predicted protein sequences for opossum CES2.2 and CES2.3. The latter sequences were predicted from BLAT studies [38,39] using the opossum CES2.1 sequence to interrogate the opossum genome but corrected following alignment with the CES2.2 (GenBank: EU019538) and CES2.3 (GenBank: EU019539) sequences. The three opossum CES2 like sequences were very similar, especially for the CES2.2 and CES2.3 sequences, which shared 94% sequence identity. In addition, the deduced amino acid sequences for opossum CES2.1, CES2.2 and CES2.3 showed 56–57% sequence identity with human CES2 and 44–47% identity with human CES1 (Figure 2; Table 2).

**Table 2:** Percentage amino acid sequence identities for human (h) and opossum (op) carboxylesterases (CES)

CES	hCES1	hCES2	hCES3	hCES4	hCES6	hCES7	opCES1	opCES2.1	opCES2.2	opCES2.3	opCES6
hCES1	100	45	42	79	32	40	63	47	44	45	40
hCES2	45	100	46	45	30	42	46	56	56	57	40
hCES3	42	46	100	41	29	40	42	46	45	45	38
hCES4	79	45	41	100	32	42	46	49	44	46	41
hCES6	32	30	29	32	100	33	31	26	28	29	45
hCES7	40	42	40	42	33	100	40	43	44	44	42
opCES1	63	46	42	46	31	40	100	48	47	48	41
opCES2.1	47	56	46	49	28	43	48	100	69	70	38
opCES2.2	44	56	45	44	28	44	47	69	100	94	37
opCES2.3	45	57	45	46	29	44	48	70	94	100	38
opCES6	40	40	38	41	45	42	41	38	37	38	100
No. of aa's	567	559	571	565	545	525	559	501	551	549	592

Several key residues in these enzymes were conserved, including (sequence numbers refer to opossum CES2) Cys94/Cys121 and Cys278/Cys289, the sites for disulfide bond formation in human CES1 [3,15,29,30,41], and active site residues responsible for the formation of acyl-ester intermediates (Ser226) and the charge relay system in catalysis (Glu343) and His (452) [3]. The deduced amino acid sequence for opossum CES2.1 was 9 residues shorter (550 residues) than for human CES2 (559 residues), and lacked the microsomal targeting sequence at the C-terminus end of the enzyme. The human CES2 C-terminal tetrapeptide sequence, His-Thr-Glu-Leu (HTEL), functions in protein retrieval from the Golgi apparatus and in CES retention in the ER lumen [12], but this is missing in opossum CES2.1 which may then influence the subcellular location and metabolic role for this enzyme in opossum liver. In contrast, opossum CES2.2 and CES2.3 sequences retain a homologous Arg-Met-Glu-Leu (RMEL) tetrapeptide sequence and these enzymes may then be localized within the liver endoplasmic reticulum. Opossum CES2.1, CES2.2 and CES2.3 sequences however share a homologous hydrophobic N-terminus signal peptide (residues 1–25, 1–24 and 1–24 respectively) with human CES2 (residues 1–26) [12].

The N-glycosylation site reported in human CES1 [42] was also found in opossum CES2.1 (86Asn-Ala-Thr) with four additional potential N-glycosylation sites observed at 274Asn-Leu-Ser, 293Asn-Lys-Thr, 376Asn-Ile-Ser and 425Asn-Ser-Ser. In contrast, opossum CES2.2 and CES2.3 lacked the N-glycosylation site at the corresponding position (85Asp-X-Thr), as did human CES2 (87Asp-X-Thr), but retained another potential site (273Asn-Leu-Ser) in common with opossum CES2.1. The two charge clamps reported for human CES1 [43] which contribute to the formation of trimers and hexamers for this enzyme are retained for one site on each of the opossum CES2 like gene products (85Arg *predicted charge clamp*188Glu) but not for the second site (86Asp...191Pro) in each case (Figure 2). The impact on the tertiary and potential quaternary structures for these opossum CES2 like enzymes remains to be determined, however it is relevant to note that human and baboon CES2 are monomers, presumably as a result of the lack of both charge clamps and the N-glycosylation site for sialic acid attachment, which contribute to subunit-subunit binding for human CES1 [43].

Other key functional residues for human CES1 and CES2 have been retained by the opossum CES2 like enzymes, including the 'Z-site' (Gly356 for human CES1), which functions in cholesterol binding; the 'side door' residues at 424Val.Met425.Phe426; and the 'gate' residue 551Phe (for human CES1), both of which have either been retained or have undergone conservative substitution. These latter sequences apparently function in facilitating the release of fatty acyl or aromatic groups, respectively, following hydrolysis [3,15,29,30]. In addition to the extensive sequence similarities observed for the human CES2 and opossum CES2Like products, there are strong similarities in predicted secondary structures for these enzymes suggesting that the αβ-hydrolase structure previously reported for human CES1 [3,15,29,30] has been predominantly retained by these enzymes (see Figure 2). There were some differences observed however for predicted secondary structures for the opossum CES2 like enzymes near key residues and these may influence enzyme function. For example, predicted helical secondary structures near the 'Z-site' and 'side door' were of different lengths for these enzymes which may influence ligand binding and product release, respectively.

### Predicted Opossum CES1 Amino Acid Sequence

The predicted amino acid sequence for opossum CES1 is shown in Figure 2 together with previously reported sequences for human CES1 and other opossum and human CES sequences [16-26]. The predicted opossum CES1 amino acid sequence (derived from N-Scan ID 1.45.030 following BLAT analysis [38,39] of the opossum genome using the human CES1 sequence) was longer (670 residues) than human CES1 (567 residues) with an additional 111 residues at the N-terminus end which did not align with the human CES1 sequence. Given the similarity of the predicted sequence for opossum CES1 with human CES1 at the N-terminus and our observations of incorrectly predicted splice sites, it is likely that opossum CES1 shares the same N-terminus start point with human CES1 and it is this sequence that is included in Figure 2.

Sequencing the RT-PCR product using primers derived from the predicted opossum CES1 sequence enabled confirmation of 750 bp of this sequence (GeneBank:EU07430).

**Figure 3:**
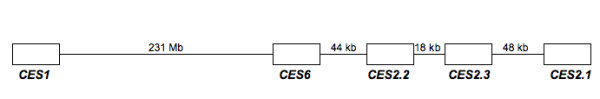
Schematic representation of the CES genes on chromosome one of the opossum.

The predicted amino acid sequence for opossum CES1 showed 63% identity with human CES1 and 46% identity with human CES2 supporting its designation within the CES1 family (Table 2). Opossum CES1 also shared several key residues with human CES1, including the active site 'triad', Ser221, Glu353 and His468 (residue numbers refer to the opossum CES1 sequence); the corresponding cysteine residues forming the disulfide bonds in human CES1 (Cys87/Cys116 and Cys273/Cys284); the microsomal C-terminus retention sequence His-Ile-Glu-Leu (HIEL); and the high-mannose N-linked glycosylation site at Asn190-X-Thr. Two other potential glycosylation sites (257Asn-Ser-Ser and 528Asn-Ile-Thr) were also observed for the opossum CES1 sequence (Fig. 3). The N-terminal microsomal signal peptide for human CES1, which retains the enzyme within the ER [12] was identical in sequence with the predicted opossum CES1 sequence, and both sequences retain 18 homologous residues in corresponding positions.

Subunit-subunit charge clamps reported for human CES1 [43] are retained for the predicted opossum CES1 protein: 72Glu *predicted charge clamp*186Arg; and 78Lys *predicted charge clamp*183Glu, suggesting that this enzyme may share the trimer-hexamer quaternary structure. The 'Z-site' glycine residue, which is a site for binding cholesterol analogues in human CES1, has been substituted in the opossum CES1 sequence (355Ala) which may reflect a change in the ligand binding properties for this enzyme. The 'side door' and 'gate' sequences for human CES1 (424Val.425Met.426Phe and 551Phe, respectively), which function in facilitating the release of fatty acid and aromatic products respectively following hydrolysis of substrate [3,15,29,30], have also undergone substitutions for these key sites, involving two of three 'side door' residues (422Leu.423Ile.424Phe) and the 'gate' residue (549Met) for opossum CES1. In each case, however, the hydropathic nature of these sites has been retained, which may reflect retention of functions for these sites. The predicted secondary structure for opossum CES1 was similar with that reported for human CES1 [3,15,29,30], although differences in helical lengths at the 'side door' and 'gate' sites were apparent (Figure 2).

### Comparisons of Opossum CES1, CES2 'Like' and CES6 'Like' Sequences with Human CES Sequences

Figure 2 and Table 2 show the amino acid sequence alignments, the predicted secondary structures and sequence identities for the predicted opossum CES1, multiple CES2Like and CES6Like proteins, as well as the six human CES gene products, previously described [16-26] (RS Holmes, J Glenn, JL VandeBerg & LA Cox: Baboon carboxylesterases 1 and 2: sequences, structures and phylogenetic relationships with human and other primate carboxylesterases, unpublished). All of the opossum and human CESs examined showed similarities in sequences and predicted secondary structures, consistent with being members of the CES αβ-hydrolase family [3,15]. Using the 3-dimensional structure reported for human CES1 as the basis for discussing structure-functional relationships [3,15,29,30], a number of common features were observed for most enzymes, including the N-terminal 'signal peptide', the cysteine residues involved in forming disulfide bridges, the active site serine, glutamate and histidine residues; and the hydrophobic regions of the 'side door' and 'gate'.

A number of differences were observed however between these family members, which have been previously reported for the human CES family members [16-24]. Human and opossum CES6 showed longer and distinct N-terminal sequences which may reflect differences in processing the signal peptide in the endoplasmic reticulum [12]. Functional charge clamps that perform key roles in maintaining the trimeric-hexameric subunit structures for human CES1 [33] were notably absent from human CES2 and CES7, while opossum CES2.2, CES2.3 and CES6, as well as human CES3 and CES6 had only one charge clamp. These may contribute to differences in the tertiary and quaternary structures for these enzymes, in particular the monomeric subunit structure for human and baboon CES2 [27] [RS Holmes, J Glenn, JL VandeBerg & LA Cox: Baboon carboxylesterases 1 and 2: sequences, structures and phylogenetic relationships with human and other primate carboxylesterases, unpublished]. The tetrapeptide C-terminal sequence (HIEL for human CES1) which performs a key role of retaining CES1 within the lumen of the endoplasmic reticulum [44] was also found (with conservative substitutions) in human CES2 (HTEL) and CES3 (QEDL), and in opossum CES1 (HIEL), CES2.2 and CES2.3 (both RMEL) but was notably absent from opossum CES2.1 and from human CES6 and CES7 C-terminal sequences. It is likely that these enzymes have different subcellular distribution profiles as compared with other CES gene products. As mentioned previously, the N-glycosylation site reported for human CES1 (79–81: NAT) is retained for the opossum CES1 (NTT) and CES2.1 (NAT) sequences, but is absent from other opossum and human CES gene products, whereas opossum CES1 (2), CES2.1 (4), CES2.2 (1) and CES2.3 (1) have other potential glycosylation sites (Figure 3). Human CES3 (105Asn-Ser-Ser107), opossum CES6 (356Asn-Ser-Thr and 455Asn-Ile-Thr), human CES6 (37Asn-Pro-Ser, 318Asn-Val-Thr, 380Asn-Ser-Thr and 465Asn-Ile-Thr); and human CES7 (281Asn-Ala-Ser, 363Asn-Lys-Ser, 463Asn-Leu-Thr and 473Asn-Met-Thr) sequences also exhibit other potential glycosylation sites which may contribute to carbohydrate binding for these proteins.

Amino acid substitutions were observed for other key regions reported for human CES1, including the Z-site Gly354 (involved in cholesterol binding) [3], which was retained for human and opossum CES2Like gene products and human CES7, but replaced in opossum CES1, human CES3 and in opossum and human CES6 sequences; the human CES1 'side door' sequence at 423Val-Met-Phe425 (proposed to regulate fatty acid residue release following hydrolysis of fatty acyl ester linkages [3]) and the 'aromatic-releasing' residue (the 'gate') at 551Phe have each undergone conservative substitutions for the opossum and human CES sequences analyzed. The impacts of these changes on the structures and functions for these enzymes remain to be determined.

### Locations for Opossum CES1, CES2 and CES6 'Like' Genes on Chromosome 1

Opossum *CES2.1 *cDNA [GenBank:EU019537] was cloned and sequenced. Using BLAT [38,39] to align the cDNA sequence to the recently published genome [40] revealed that the gene is localized on chromosome 1. The identification of this gene as *CES2.1 *is based upon the identity of the corresponding predicted protein with the deduced amino acid sequence for opossum CES2.1 (Figure 2). The BLAT studies localized this gene on chromosome 1 at nucleotides 677,900,919–677,927,002 on the negative strand with 99.9% identity (Table 1; Figure 3). Two further CES2 'like' genes were identified on this chromosome at nucleotides 677,773,395–677,808,416 (*CES2.2 *gene) and 677,826,454–677,852,862 (*CES2.3 *gene), also on the negative strand. Consequently, all 3 CES2 like genes are localized within 155 kb of DNA on chromosome 1 (Figure 3). The predicted proteins from the genes (designated as CES2.2 and CES2.3) were highly similar with each other (94% identical) and with opossum CES2.1 (~70% identical) (Table 2).

The human CES1 amino acid sequence was used for BLAT interrogation of the opossum genome, generating an opossum *CES1 *homologue gene and a predicted amino acid sequence for this enzyme. The responsible gene (*CES1*) was located at nucleotides 446,224,784–446,256,371 on chromosome 1 with a span of 31588 nucleotides on the negative strand (Table 1; Figure 3). This protein was more similar with human CES1 (63% identity) than with human CES2 (46% identity) (Table 2). Another study was conducted using human CES6 for BLAT interrogation of the opossum genome and evidence obtained for a region of sequence similarity, located within 44 kb of the *CES2.2 *gene (Table 1; Figure 3). A predicted protein sequence for opossum CES6 was obtained and compared with other opossum and human CES gene product sequences in Figure 2. These results strongly suggest that there are three *CES2 *'like' genes which are 92 kb apart on opossum chromosome 1; a *CES6 *'like' gene, which is 44 kb closer to the *pter *region than the *CES2 *gene complex; and a *CES1 *'like' gene, which is further upstream on chromosome one (Figure 3). The RT-PCR studies reported earlier for opossum *CES1*, *CES2, CES2.2 *and *CES2.3 *genes confirm the existence, expression and sequences for CES coding regions for these opossum genes.

Human CES3 and CES7 amino acid sequences were also used to interrogate the opossum genome using the BLAT method [38,39], however the results were inconclusive and further molecular genetic analyses will be required to establish the presence or otherwise of these CES genes on the opossum genome.

### Phylogeny and Divergence of Mammalian CES Sequences

**Figure 4:**
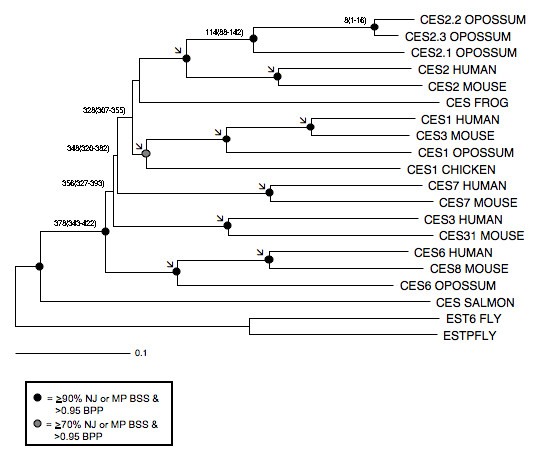
**Phylogenetic tree of selected vertebrate CES amino acid sequences.** Each branch of the tree is labeled with the gene name followed by the species name. Shown is the neighbor joining (NJ) tree based on JTT+gamma corrected distances. Nodes with strong NJ and maximum parsimony (MP) bootstrap support (BSS) and Bayesian posterior probability (BPP) support are highlighted with black and grey dots (see legend at bottom). Black arrows indicate nodes constrained in the MULTIDIVTIME analysis (see Materials and Methods for details). Divergence time estimates (MY) and 95% confidence intervals are given for early branching events that gave rise to the modern CES gene families, and the more recent duplication events that led to the three marsupial CES2 family members.

A phylogenetic tree (Figure 4) was estimated using a progressive alignment of 6 human CES amino acid sequences with the following opossum CES sequences: CES2.1 (derived from sequencing a full-length cDNA); CES1, CES2.2 and CES2.3 (derived from BLAT interrogations of the opossum genome and from sequencing cDNA clones of RT-PCR products for these genes); and CES6 (derived from blat interrogation of the opossum genome using human CES6) [39,40]. We also included other vertebrate CES homologues including chicken, frog and salmon, in addition to two divergent fly sequences as outgroup sequences.

The amino acid distance tree (Figure 4) shows a cluster of five main groups consistent with *CES1*, *CES2, CES3*, *CES6*, and *CES7 *gene families being products of ancestral gene duplication events, and indicates that all families arose prior to the divergence of therian mammals ~173–193 MYA [45-47]. This is consistent with a previous report for mammalian CES genes [28] and also with other studies that showed mammalian *CES1*, *CES2 *and *CES7 *are members of distinct but related gene families [2,7] [RS Holmes, J Glenn, JL VandeBerg & LA Cox: Baboon carboxylesterases 1 and 2: sequences, structures and phylogenetic relationships with human and other primate carboxylesterases, unpublished]. Phylogenetic trees based on maximum parsimony and Bayesian methods produce very similar results, with similarly high bootstrap and posterior probability support for the distinct CES clusters observed in the distance tree (Fig. 4). No strong support for early branching patterns emerged from any of the different phylogenetic analyses, suggesting the different gene families arose during a short period of time.

Opossum *CES1 *is an member of the gene *CES1 *family which has been previously shown to include other mammalian CES1 gene products from human, baboon, rat and mouse, as well as human CES4 [2,27] [RS Holmes, J Glenn, JL VandeBerg & LA Cox: Baboon carboxylesterases 1 and 2: sequences, structures and phylogenetic relationships with human and other primate carboxylesterases, unpublished]. Opossum CES2.1, CES2.2 and CES2.3 gene product sequences, however, show phylogenetic association within the eutherian CES2 group, which includes multiple CES2-like genes from mouse and rat (mouse was included as a representative of this lineage in our analyses) [48-51]. Eutherian *CES3, CES6 *and *CES7 *genes form distinct lineages, each originating during the early, pre-mammalian radiation of the CES gene family. Human CES3 shares 46% identity with human and opossum CES2 gene products (Figure 3 and Table 2). Human and opossum *CES6 *and human *CES7 *share an average 40% and 42% identity respectively, with other human and opossum CES products (Table 2). Our inclusion and phylogenetic placement of chicken, frog and salmon sequences (Fig. 4) is consistent with the view that the early CES divergence events occurred during the early stages of tetrapod evolution, but following divergence from bony fishes, which diverged much earlier from the common ancestor of all tetrapod CES sequences.

### Estimates of Evolutionary Dates for CES Gene Duplication Events

We estimated the times of divergence for the CES gene family and its members using a relaxed clock approach [52,53], an amino acid alignment, and three major calibration points: mouse-human, eutherian-marsupial, and synapsid-diapsid divergence times estimated from the literature. Our results suggest that the earliest branching events that produced the extant mammalian CES gene families occurred between 378 and 328 MYA (Fig. 4). Divergence dates for the earliest splits coincide with the divergence time of tetrapods approximately 350–360 MYA based on both molecular and paloeontological data [45,54,55] and are consistent with these events predating the tetrapod divergence and post-dating the tetrapod-bony fish split based on two observations: the placement of chicken and frog CES sequences in different CES gene families, and the early divergence of salmon CES-like sequence from the tetrapod sequences (Fig. 4). Based on these findings, it is proposed that several ancient *CES *gene duplication events took place prior to the origin of mammals, generating five ancestral genes for *CES1, CES2, CES3, CES6 *and *CES7*, which have all been retained over the past 180–230 million years of mammalian evolution. Subsequent gene duplications apparently took place prior to (~114 MYA) and during (~8 MYA) the evolutionary diversification of extant marsupials [55], generating three *CES2-*like genes in the opossum genome, in contrast to a single *CES2 *gene found in some eutherians.

## Conclusion

We have conducted the first study of marsupial *CES *genes using BLAT [38,39] interrogation of the recently published opossum genome sequence [40] and studied gene expression by RT-PCR, and sequenced opossum *CES1 *and *CES2Like *cDNAs. We found that opossum *CES *genes are located in two regions on chromosome 1, with a *CES1like *gene ~231 million bp upstream of a *CES2/CES6 *gene cluster, which comprises three *CES2like *genes (designated as *CES2.1*, *CES2.2 *and *CES2.3) *and a *CES6like *gene. An opossum *CES2.1 *cDNA was isolated and sequenced and the predicted protein sequence shown to be more similar to human CES2 among six human CES sequences previously reported. RT-PCR expression studies demonstrated that the opossum *CES1*, *CES2.2 *and *CES2.3 *genes are transcribed in opossum liver and intestine, whereas the opossum *CES2.1 *gene was expressed in liver and not intestine. Amino acid sequence alignments for these opossum gene products with human CES isozymes demonstrated considerable conservation of key residues involved in catalysis, organelle localization, quaternary structure and ligand binding, and retention of the α-β secondary structures previously reported for human CES1 [3,15,29,30,43]. A phylogenetic study was conducted using the opossum CES sequences and amino acid sequences for other vertebrate CES isozymes. The pattern and timing of the CES gene family radiation suggests that a number of gene duplication events occurred prior to the appearance of mammals [45,46], between 330–370 MYA generating ancestral genes for *CES1, CES2*, *CES3*, *CES6 *and *CES7 *in the common ancestor of marsupial and eutherian mammals. Further gene duplications for *CES2 *occurred in the lineage leading to modern marsupials, resulting in three *CES2*-like genes within a 154 kb region on opossum chromosome 1.

## Methods

### Cloning and sequencing CES2 cDNA

A 421 bp opossum cDNA (GenBank: DR038241) which exhibits similarity to CES of other species is in the public database. To study opossum *CES *genes, primers were designed using this DNA sequence to amplify a full-length opossum *CES *cDNA from liver RNA by the RNA ligase-mediated rapid amplification of 5' and 3' cDNA ends (RACE) method [56]. The GeneRacer kit (Invitrogen, Carlsbad, CA) was used for RACE according to the manufacturer's instructions. For 5' RACE, the GeneRacer 5' primer from the kit and a gene-specific primer (5'-acccaccgaagggcagccacctgat-3') were used in the first round of amplification, then the GeneRacer 5' nested primer from the kit and a gene-specific nested primer (5'-gcctgacgcatactcatccccagtgct-3') were used in the second round of amplification. For 3' RACE, one round of PCR with the GeneRacer 3' primer from the kit and a gene-specific primer (5'-tgtggccatccttcctggcatgctt-3') was sufficient to obtain a RACE product. RACE PCR products were cloned into the pCR4-TOPO vector (Invitrogen). Sequencing was performed using the BigDye Terminator v3.1 Cycle Sequencing Kit (Applied Biosystems, Foster City, CA), and nucleotide sequences were analyzed on an ABI Prism 3100 DNA sequencer (Applied Biosystems).

### RT-PCR Expression Studies and Sequencing of RT-PCR Products

Total RNA was isolated from livers and small intestines of opossums using the TRI Reagent (Molecular Research Center, Cincinnati, OH), and treated with DNase I from the Turbo DNA-free kit (Applied Biosystems) according to the manufacturer's protocol to remove contaminating DNA from the RNA preparations. DNase I-treated RNA was reverse transcribed into cDNA using a High Capacity cDNA Reverse Transcription kit (Applied Biosystems). Control RT-PCR reactions were conducted with DNase-treated liver and intestine RNA in the absence of reverse transcriptase. Liver and intestinal cDNAs were used as templates in RT-PCR to analyze expression of the predicted/cloned CES genes. Primers for the *CES *genes were as follows. For the *CES1 *gene, the forward primer was 5'-attcaggggaagcagtcctc-3' and the reverse primer was 5'-tgccatgatgctggaattgt-3'. For the *CES2.1 *gene, the forward primer was 5'-tgcctcgttgccaatctatctgcttgtg-3', and the reverse primer was 5'-tcagtagtcatgatctcccaatag-3'. For the *CES2.2 *gene, the forward primer was 5'-catttgtggctcagcttctgct-3' and the reverse primer was 5'-gggcaggaataacaaacatccag-3'. For the *CES2.3 *gene, the forward primer was 5'-ttgtctgcatcccagaatgtgata-3' and the reverse primer was 5'-gggcaggaatagcaaacatcaaa-3'. The CES2.2 and CES2.3 cDNAs are 97% identical to each other, therefore primers were selected such that mismatch at the 3' end of primers would allow only the target gene to be amplified. Primer sets for the four CES genes span introns, and the size of the PCR products and the absence of PCR products in the control studies demonstrates that they were not amplified from genomic DNA.

### In silico Studies of Opossum CES Genes

BLAT (BLAST-Like Alignment Tool) *in silico *studies were undertaken using the UC Santa Cruz web site with the default settings (Assembly:2006; Query: BLAT's guess; Sort Output: query score; Output type: hyperlink) [38,39]. Opossum CES2.2 and CES2.3 genomic alignments were confirmed using Ensembl (Ensembl release 45, MonDom5 assembly, June 2007) [57,58]. The following UniProtKB/Swiss-Prot Database sequences were used to interrogate the opossum genome sequence [38]: human sequences CES1 (P23141), CES2 (O00748), CES3 (Q6UWW8), CES4 (Q8TDZ9); CES6 (Q6UX55) and CES7 (Q96DN9) (Table 1); as well as the opossum CES2 sequence reported in this paper. Genome locations and predicted protein sequences were obtained for each CES sequence used and the results for those regions showing high (>70%) levels of identity with the human CES gene products or with opossum CES2.1 (full identity) were examined and compared with opossum and human CES sequences using the SIM-Alignment tool for Protein Sequences [57,58] (see Table 1). Protein sequences were generated *in silico *for opossum *CES *genes using the UC Santa Cruz Genome Browser [38,39] and human CES1, CES2 and CES6 sequences in BLAT analyses of the opossum genome. Sequences for opossum CES1, CES2.2 and CES2.3 were aligned with the translated sequences for the corresponding RT-PCR products to ensure identity in each case.

### Phylogenetic Studies and Sequence Divergence

Phylogenetic trees were constructed using an amino acid alignment from a ClustalW-derived alignment of mammalian, chicken, frog, salmon CES protein sequences, obtained with default settings [59,60]. Two fly (*Drosophila melanogaster*) CES sequences were included as outgroups. Alignment of ambiguous regions, including the amino and carboxyl termini, were excluded prior to phylogenetic analysis, yielding an alignment of 367 residues. Amino acid distance trees were built in PHYLIP (v. 3.57) using JTT+gamma corrected distances and the neighbor joining algorithm [61]. Maximum parsimony trees were constructed in PAUP 4.0b [62] using heuristic searches (50 iterations with random addition of taxa). Bootstrap results for each method were based on 100 iterations under similar search criteria. A Bayesian analysis was performed in Mr.Bayes (vers. 3.1.2) [63] under the following search criteria: two independent runs of 1 million generations, with 4 independent chains sampled every 100 generations, under a mixed amino acid model, with a burnin set at 100,000 generations. The average standard deviation of split-frequencies between the two runs was <0.001.

Divergence dates were obtained with the programs ESTBRANCHES and MULTIDIVTIME [50,51], assuming the NJ topology (Fig 4). The salmon CES sequence was included to root the tree. Branch lengths and variance-covariance matrices were estimated assuming the JTT model of amino acid replacement, and these branch lengths were used to estimate divergence times in MULTIDIVTIME. We used the following calibration points: (1) 84 and 99 MYA for the minimum and maximum ages, respectively, for each of the primate/rodent splits in Fig. 4 based on 95% confidence intervals of published molecular divergence estimates [64], (2) 173 and 193 MYA for the minimum and maximum ages, respectively, of each eutherian/metatherian split in Fig. 4 based on published molecular divergence estimates [45-47], and (3) 300 and 320 MYA for the minimum and maximum ages, respectively, for the chicken/mammal CES1 divergence, based on a conservative 20 MYA window surrounding the well-constrained synapsid/diapsid split at 310 MYA [46]. The prior for the root was set at 360 MYA, on the basis of previous paleoontological and molecular estimates for the age of tetrapods [46,54].

#### Predicted Secondary Structures

Secondary structure analyses were undertaken using the PSIpred tools provided by Brunel University [65,66].

## Abbreviations

cDNA: complementary DNA; RT-PCR: Reverse Transcription-PCR; CES: Carboxylesterase (E.C.3.1.1.1); BLAST: Basic Local Alignment Search Tool; BLAT: BLAST-Like Alignment Tool; MYA: Millions of years ago

## Authors' contributions

RH and JC conceived the study. RH, JC, LC, WM and JV participated in its design and coordination. JC carried out cloning, DNA sequencing and RT-PCR studies. RH performed the BLAT and alignment studies. WM and RH carried out the phylogeny studies. RH and JC outlined the manuscript together and RH drafted the manuscript. All authors have read and approved the final manuscript.

## Supplementary Material

Additional file 1 Controls for RT- PCR studies examining the expression of *CES *genes in opossum. The data presents RT-PCR controls showing that there were no RT-PCR products produced in the absence of reverse transcriptase (figure S1) compared with the results repeated here showing the RT-PCR products were formed when the experiment was conducted in the presence of reverse transcriptase (figure S2). Click here for file
