# Supplementary material for: Opossum carboxylesterases: sequences, phylogeny and evidence for CES gene duplication events predating the marsupial-eutherian common ancestor
Source: BMC Evol Biol. 2008 Feb 20;8:54. doi: 10.1186/1471-2148-8-54 (PMC2266714; doi:10.1186/1471-2148-8-54)

**Figures S1A and S1B**

**Legend:**

Liver and intestinal DNase I-treated RNAs were examined in the absence of reverse transcriptase (Figure S1A), whereas RT-PCR reactions were performed with DNase I-treated liver RNA in the presence of reverse transcriptase (Figure S1B). One microliter of the RT reaction was used to amplify *CES* cDNAs by PCR. For Figure S1A, Lanes 2 and 3 are controls for RT-PCR products amplified from liver (L) and intestine (I) cDNAs for *CES1* gene; lanes 4 and 5, controls for RT-PCR products from liver (L) and intestine (I) cDNAs for *CES2.1* gene; lanes 6 and 7, controls for RT-PCR products from liver (L) and intestine (I) for *CES2.2* gene; and lanes 8 and 9, controls for RT-PCR products from liver (L) and intestine (I) for *CES2.3* gene. For Figure S1B, a repeat is shown of the RT-PCR studies using liver DNase I-treated opossum liver RNA in the presence of reverse transcriptase for *CES1, CES2.1, CES2.2 and CES2.3* opossum genes. M shows the DNA size ladder.


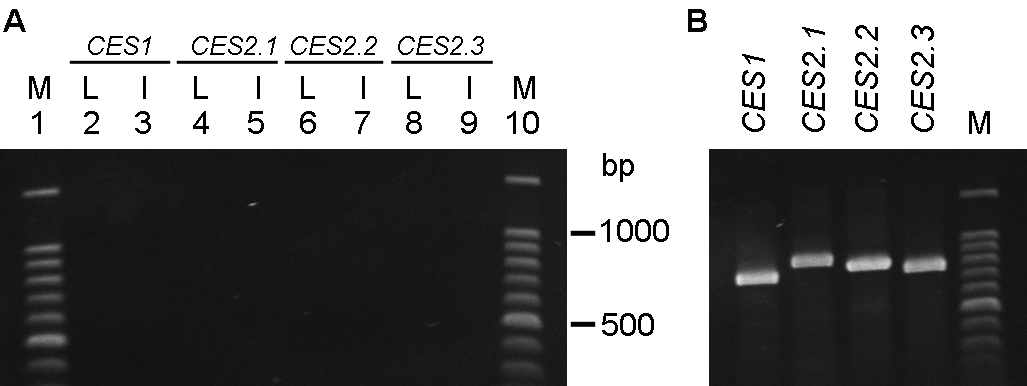

Supplement: Additional file 1 — Controls for RT- PCR studies examining the expression of CES genes in opossum. The data presents RT-PCR controls showing that there were no RT-PCR products produced in the absence of reverse transcriptase (figure S1) compared with the results repeated here showing the RT-PCR products were formed when the experiment was conducted in the presence of reverse transcriptase (figure S2). [file 1471-2148-8-54-S1.DOC]
